# HOX Genes in High Grade Ovarian Cancer

**DOI:** 10.3390/cancers11081107

**Published:** 2019-08-03

**Authors:** Praveena Idaikkadar, Richard Morgan, Agnieszka Michael

**Affiliations:** 1 Postgraduate Medical School, Faculty of Health and Medical Sciences, University of Surrey, Guildford GU2 7YS, UK; 2 Institute of Cancer Therapeutics, Faculty of Life Sciences, University of Bradford, Bradford BD7 1DP, UK

**Keywords:** HOX, ovarian cancer

## Abstract

HOX genes are highly conserved members of the homeobox superfamily that have a crucial role in determining cellular identity. High grade ovarian cancer is the most lethal gynaecological malignancy. Our understanding of the role of HOX genes in the oncogenesis of ovarian cancer is evolving, and here we review their dysregulated expression patterns, their function in cell survival and invasion, their potential uses as biomarkers, and ways in which HOX genes are being targeted with new and existing drugs.

## 1. Introduction

HOX genes are a member of the homeobox superfamily, with “homeotic” or “HOX” genes coding for transcription factors that were originally recognized for their role in embryogenesis in the *Drosophila melanogaster* fruit fly [1]. There are now known to be 39 HOX genes in mammals, grouped into four paralogous clusters termed A to D on four different chromosomes (7, 17, 12 and 2 respectively). They are numbered 1 to 13 based on their 3′ to 5′ position [1].

HOX proteins can function as monomers, homodimers, heterodimers or heterotrimers, and the affinity and specificity of their binding to DNA can be modulated by binding proteins of the three amino acid loop extension (TALE) family of co-factors, especially Pre-B cell leukaemia transcription factor (PBX) and myeloid ectopic viral integration site (MEIS) [2].

The roles of HOX genes are wide and varied, including embryonic development and patterning of the anterior to posterior axis, and there is growing evidence that they have a pivotal role in oncogenesis in a number of tumour types, including ovarian cancer [1,3]. Their effects are complex, with many genes having overlapping or redundant functions and sometimes contradictory effects in different tissue types.

## 2. The Role of HOX Genes in Cancer

A link between HOX genes and cancer was first evident in haematological malignancies, but since this discovery they have now been implicated in the oncogenesis of a variety of solid organ malignancies with broadly HOX A genes often showing altered expression in breast and ovarian cancers, HOX B genes in colon cancer, HOX C in prostate and lung cancers and HOX D in colon and breast cancers [3].

HOX genes can act as both oncogenes and tumour suppressors but are usually pro-oncogenic in a more supportive role, both at the cellular level, for example by driving cell proliferation and preventing apoptosis, and also at the tumour level, for example by promoting angiogenesis, metastasis and treatment resistance [2].

The mechanisms of HOX gene action are complex and not fully elucidated, and it is not within the scope of this article to give a detailed mechanistic picture. However, broadly, the protein products of HOX genes act as both positive and negative transcription factors that can interfere with various cell signaling pathways, and thus upset the delicate balance of regulatory mechanisms that prevent carcinogenesis [3]. Abate-Shen proposed that the effects of HOX genes may be dependent on their expression levels, as well as changes in tempero-spatial patterning [4], where HOX gene expression in a tumour of a specific tissue is different from the expression seen in the normal tissue at a given time or in a different location.

## 3. HOX Genes in Ovarian Cancer

HOX genes are expressed in both normal ovarian surface epithelium (OSE) and also in high grade ovarian carcinomas, and their differential expression in these tissues has been investigated in a number of studies [5,6,7,8,9,10], with a wide range of different HOX genes showing dysregulation (see Table 1). The vast majority of studies on HOX genes and their role in ovarian cancer have focused on high grade cancers, predominantly high grade serous carcinoma, and there is a lack of data regarding low grade ovarian cancer. This review will concentrate on HOX genes in high grade ovarian/fallopian tube cancer.

**Figure 1:**
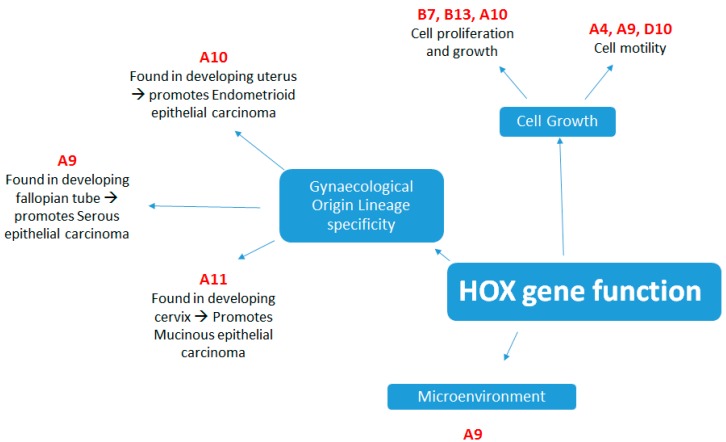
The main functions of HOX genes in the oncogenesis of ovarian carcinoma.

In the female reproductive system four HOX genes, HOXA9, HOXA10, HOXA11 and HOXA13 are expressed along the Mullerian duct axis. These tandemly arranged HOX genes are expressed uniformly in embryonic development, although in adults their expression becomes spatially restricted to particular organs. HOXA9 becomes expressed in the fallopian tubes, HOXA10 is expressed in the developing uterus, HOXA11 in the lower uterine segment and cervix and HOXA13 in the upper vagina [11]. It is thought that inappropriate expression of these genes is an early step in epithelial ovarian neoplasia as they induce aberrant epithelial differentiation. A landmark paper by Cheng et al. [12] showed that these HOX genes were not expressed in normal adult OSE but were responsible for inducing lineage specificity in ovarian cancers, with HOXA9 promoting a papillary serous phenotype, HOXA10 endometrioid and HOXA11 mucinous ovarian adenocarcinoma (see Figure 1). HOXA7 was also non-specifically expressed in all sub-types of high grade ovarian carcinoma and the presence of HOXA7 was associated with a lower grade phenotype. The association between HOXA10 and endometroid epithelial ovarian cancer was confirmed in a further study from a different group, who also showed repression of HOXA10 transcription through Wilms tumour 1 (WT1) binding to a repressor site [13].

A comprehensive analysis of high grade serous (HGS) ovarian cancer specimens in the TCGA database found a dysregulation of HOX proteins in a subgroup of samples termed the “mesenchymal” group, characterized by high expression of HOXB2, B5 and B8 [9]. A further comprehensive analysis of HOX gene expression in several cell lines of different histological subtypes of ovarian cancer (HGS, endometroid and clear cell) showed marked HOX gene dysregulation which varied by cell line and sensitivity to platinum-based chemotherapy. The study went on to examine samples from 73 patients with HGS ovarian cancer and found that a 5-gene “HOX signature” of HOX A13, B6, C13, D1 and D13 was associated with poor survival [7]

A small study by Hong et al. [5] showed 11 HOX genes (A7, B3, B4, B6, C10, C11, D1, D3, D10, D11, D13) were either up- or down-regulated in ovarian cancer tissues compared to normal ovarian tissue, and HOXB4 had a significantly higher expression at the RNA and protein level. A large microarray gene expression analysis of 65 ovarian samples comparing non-malignant and malignant ovarian tissue showed a significant up-regulation of HOX A5, A9, B2, B5, B6, B7 and D1. There was a significant downregulation of HOXC6 [14]. This was corroborated by the results of a further study by Tait et al., also showing reduced expression of HOXC6 in serous ovarian cancer [15]. A study by Yamashita et al. [10] showed 16 HOX genes were over-expressed with HOXB7 and B13 showing near exclusive expression in cancer cells and contributing to a malignant phenotype.

The very high degree of heterogeneity in ovarian cancer, and especially in HGS carcinoma, goes some way to explaining some of the results and differences between various studies. Common patterns of expression are described below and classified by the likely function of the HOX genes and proteins in this common and very aggressive malignancy.

## 4. HOX Function in Ovarian Cancer

### 4.1. Tumour Growth

HOX genes can function as both oncogenes and tumour suppressors, and many studies have shown an effect upon cell growth and proliferation. A study by the Naora group [16] showed overexpression of HOXB7 in immortalized OSE cells up-regulated basic fibroblast growth factor (albeit intracellularly) and enhanced cell proliferation. This correlated with a study showing that knockdown of B7 and B13 with overexpression of antisense, suppressed transwell invasion of SKOV-3 cells [10]. Another study demonstrated a role for HOXB13 in cell growth both in vitro and in vivo, B13 knockdown with siRNA in cell lines reduced cell proliferation and ectopic expression of B13 in p53 negative mice promoted tumour growth via the ras pathway [17].

Several studies have shown that HOXA10 can increase the growth and invasion of ovarian cancer cells [18,19,20], and that endometroid, clear cell and mucinous tumours (but not serous) express significantly higher levels of HOXA10 compared with normal ovarian tissue. These studies also showed a worse overall survival in patients whose tumours had high levels of HOXA10.

### 4.2. Cell Motility and Invasion

HOX genes have been shown to have a role in the invasive properties of ovarian cancer cells, especially the HOX A class. This is particularly important, as many patients with ovarian cancer present at a late stage with widely disseminated disease. Seeding of tumour cells onto the peritoneum is a potentially attractive early target for reducing the lethality of ovarian cancer.

HOXA4 seems to have a contradictory role in cell invasion. The Auersperg team [21] found that HOXA4 expression was increased in invasive compared to noninvasive tumours, although it was acting as a tumour suppressor by reducing cell migration. HOXA4 knockdown increased OVCAR-3 and 8 cell migration and spreading but did not increase invasion by the Matrigel assay. The putative mechanism for this inhibitory effect involves the modulation of cell-cell adhesion via β1 integrin, although it was noted that the RNA level of β1 integrin was not affected, only the protein level, and the hypothesis was that HOXA4 was acting by some unknown post-transcriptional modulation. These results are supported by a study showing siRNA mediated knockdown of HOXA4 promoted normal OSE cell motility, but only in the presence of EGF [22].

Similar reports of the action of HOXA9 have shown that HOXA9 suppression via microRNA-196b (which is overexpressed in recurrent compared to primary epithelial ovarian cancer) increased the invasiveness of SKOV-3 cells in vitro [23]. Another study using 7 cell lines and 68 primary tumours has proposed a similar function for HOXD10 [24], and revealed that HOXD10 was inhibited by miRNA-10b, resulting in increased migration and cell invasion via matrix metallopeptidase 14 and ras homolog family member C.

### 4.3. Immune System Mediation

The Naora team conducted a series of experiments [25,26] where they showed that expression of HOXA9 by cancer cells increased their ability to recruit and condition local peritoneal macrophages to exhibit a more aggressive phenotype that suppresses anti-tumour immune responses and become “tumour associated macrophages” (TAMs). This was mainly achieved via TGF-β2 and CCL2 signaling, which also stimulates fibroblasts and mesenchymal stem cells to support cancer cell growth and angiogenesis. Finally, HOXA9 was also able to stimulate cancer cells to attach to peritoneal mesothelial cells via p-cadherin encoded by the CDH3 gene. HOXA9 expression was associated with poor overall survival in patients. The same group showed that HOXA10 can also stimulate OSE cells to grow and interact with the extracellular matrix and mesothelial cells in the omentum, promoting cell adhesion [27].

Analysis of The Cancer Genome Atlas (TCGA) dataset [9] revealed that at least a subset of high grade serous ovarian cancers were immune-reactive and some teams have used newer technologies to identify target antigens of interest that stimulate an immune reaction. The Naora group used serological identification of antigens by recombinant expression cloning (SEREX) technology to screen the plasma of ovarian cancer patients against tumour cDNA expression libraries, for antibodies to potential novel tumour antigens [16]. They found that HOXB7 was a valuable target with 13/39 ovarian cancer patient serum reacting to HOXB7 protein, compared to 1/29 healthy controls. They went on to show markedly higher B7 expression in tumours compared to normal OSE and forced expression of B7 in immortalized OSE cells enhanced cell proliferation (See also Section 4.1.

The same group showed similar findings with HOXA7; 16/24 patients with moderately differentiated serous ovarian carcinomas were found to have significantly more anti-HOXA7 antibodies compared to healthy controls. However, interestingly, patients with poorly differentiated serous carcinomas did not show serologic reactivity, whilst 68% of patients with benign serous cystadenomas did [28].

## 5. Targeting HOX Genes

### 5.1. Targeting Co-Factors

The literature shows a great deal of functional redundancy in HOX genes, with many having overlapping or contradictory roles. It has also been difficult to create effective small molecule inhibitors of individual HOX proteins. HOX protein binding to DNA is modulated and enhanced by the binding of co-factors, especially PBX and MEIS. Targeting this interaction is a pragmatic way to affect the function of multiple HOX genes as it is mediated by a highly conserved hexapeptide sequence in HOX proteins [2]. PBX and MEIS are extensively expressed in ovarian carcinomas, but only MEIS 1 and 2 are expressed in normal OSE [29], potentially allowing for good drug tumour specificity for an inhibitor of PBX.

Our group has developed a cell-penetrating peptide, HXR9, which contains the hexapeptide sequence and acts as a competitive antagonist of HOX/PBX binding. We have tested this drug in two ovarian cancer cell lines, SKOV-3 which shows highly dysregulated HOX expression and OV-90, which does not. HXR9 was able to induce apoptosis and retard tumour growth in vivo for the SKOV-3 cells but not the OV-90 cells [30]. Subsequent work on additional cell lines also demonstrated sensitivity to HXR9, but not to a control peptide CXR9, and HXR9 showed synergy with Cisplatin chemotherapy in both cell lines and mouse models [7]. These results have been re-capitulated in other tumor types including melanoma, renal cancer and breast cancer.

### 5.2. Long Non Coding RNA

Only 2% of the human genome codes for proteins whilst the majority of open reading frames are for non-coding RNA, which can still regulate gene expression and play a critical role in multiple physiological and disease processes. Non-coding RNA can be split into micro RNA (miRNA) and long non coding RNA (lncRNA) depending on their sequence length. The expression and function of lncRNA in ovarian cancer cells versus normal ovarian tissue was examined in a study which showed 2870 lncRNAs were differentially expressed with a “HOX cluster” that was highly dysregulated [31].

Specific lncRNAs have been investigated for their individual roles in ovarian cancer. For example, HOXA11-AS has two exonic variants and the minor allele rs17427875 (A > T) was relatively more common in cases of serous epithelial ovarian cancer (although this association did not reach statistical significance). Ectopic expression of HOXA11-AS in cancer cells reduced cell survival, proliferation, migration and invasion in vitro, and reduced tumour growth in mouse models, the T allele more so than the A allele [32].

Another group showed that the lncRNA GAS5 is downregulated in ovarian cancer, and works to inhibit miRNA-196a-5p, which in turn down-regulates HOXA5 that acts as a tumour suppressor in this context. Strategies to decrease HOXA5 expression (by si-GAS5, miR-196a-5p mimic or si-HOXA5) increased cell growth and decreased apoptosis both in vitro and in vivo. Lower GAS5 expression in tumors was associated with a larger tumor size and advanced FIGO stage [33].

HOX transcript antisense RNA (HOTAIR) is a lncRNA found in the HOXC cluster that regulates the HOXD cluster and has been extensively studied in ovarian cancer. It has been shown to be overexpressed in epithelial ovarian cancers, and multivariate analysis has shown that HOTAIR expression is an independent prognostic factor for overall survival (CI 1.04–5.31, *p* = 0.04) [34]. In the same study, suppression of HOTAIR reduced cell migration and invasion and the volume and pattern of tumour growth in vivo. They showed that HOTAIR acts via regulation of MMP3, MMP9, E-cadherin, vimentin and snail.

There have also been reports of a link between HOTAIR and DNA damage response, particularly that induced by platinum chemotherapy, and that HOTAIR could mediate platinum resistance. One study showed increased HOTAIR levels in recurrent platinum resistant ovarian tumours versus primary ovarian cancers [35] and that upregulation of HOTAIR induced platinum resistance by increasing DNA damage response pathways including the NF-κB pathway. The same team have developed a peptide which can block the binding of HOTAIR to its partner EZH2. In HOTAIR over-expressing ovarian cancer cell line assays, this drug has been able to decrease invasion and increase chemotherapy sensitivity. Treatment of mice with platinum-resistant ovarian tumour xenografts reduced HOTAIR expression, decreased tumour size and improved survival. Interestingly the drug had no effect upon platinum sensitive cells [36].

## 6. HOX Genes as Biomarkers

Most ovarian cancers present at an advanced stage when the disease is widely disseminated and likely incurable. Therefore, an early diagnostic biomarker would greatly improve survival. HOX genes are an attractive biomarker as they are often exclusively expressed in cancer cells or highly dysregulated, and expressed at an early stage of the disease. Large studies, including genome wide association studies, have identified HOX genes as playing a key role in ovarian cancer risk [6]. The Naora team [16] undertook a systematic search for a diagnostic antigen via SEREX methodology (discussed previously) and found HOXB7 was a potential diagnostic antigen with 13/39 ovarian cancer patients producing antibodies against it versus 1/29 healthy females. The same team found similar results in serous ovarian carcinoma with HOXA7 [28].

An international study by Jacobs et al. [37] investigated HOX gene promoter methylation in normal endometrium as a diagnostic biomarker for the presence of ovarian cancer. They looked at matched sets of hysterectomy specimens, one set taken from patients with ovarian cancer having primary debulking surgery and one set from patients having a hysterectomy for non-malignant reasons, most commonly fibroids and endometriosis. The samples were all analysed by a histopathologist to ensure that the endometrium was normal and no detached ovarian cancer cells had contaminated the endometrium. The study found that in the patients with ovarian cancer, the normal endometrial tissue showed hypermethylation of HOXA9 and HOXA11 but this was not the case in patients without cancer. This difference was highly significant and the methylation status was able to predict the presence of cancer.

HOX genes can also act as prognostic biomarkers to stratify high and low risk patients. A study in clear cell carcinomas [20] showed that HOXA10 was significantly overexpressed and that its expression was positively correlated with poor survival. Another study showed high HOXB5 and B8 expression was linked with poor survival when looking at patient matched effusions and solid lesions [38]. HOXC8 has also been shown to be associated with poor survival [39]. A much larger study using TCGA data (630 patients) showed that HOXA10 and B3 were associated with poor survival and HOXC5 was associated with longer survival [40]

Individual genes show variability in different cell lines and histological subtypes of ovarian cancer, therefore a gene panel may be a more useful way to examine HOX gene expression. In a study by Michael et al. [7] a five-gene signature (HOXA13, B6, C13, D1 and D13) was predictive of poor survival. The same study also found a difference in HOX gene expression between platinum sensitive and resistant tumours in paired cell lines, with the resistant tumours showing a higher expression of HOXB3, B4 and B9. This could be used to stratify patients for drug selection. A clinical study showed that expression of HOXA4/HOXB3 in patients with HGS ovarian cancer was significantly associated with shorter progression free survival (PFS; HR 8.89, 95% CI = 2.09–37.74, *p* < 0.001), and that this could reflect a role for these genes in mediating platinum resistance [41].

In addition to gene expression, the epigenetic changes in HOX genes are being studied as biomarkers, particularly methylation status. A large study by Fiegl et al. found that the methylation status of HOXA10 and A11 was the best discriminator between neoplastic and normal tissue, and that HOXA11 methylation status was independently associated with poor outcome [42]. Cheng et al. confirmed this, showing that HOXA10 was significantly hypomethylated in ovarian cancer leading to increased expression [43]. A large study looking at global methylation changes in twenty ovarian cancer specimens, grouped into different histological grade and stages [44] and compared to controls, found widespread DNA hypermethylation, especially in HOX (including HOXD3, A9 and A10) and other developmental genes, even in the low grade tumours. They also found significant DNA hypomethylation, but only in the high grade tumours. Further work has confirmed the importance of HOXA9, one study found the cumulative methylation score for three genes (HOXA9, RASSF1A and OPCML) was significantly associated with tumour stage, especially when combined with CA-125 level [45]. A further study found that HOXA9 was hypermethylated in 51% of tumour samples, particularly earlier stage carcinomas which were mostly of the endometriod histological subtype [46].

## 7. Conclusions

HOX genes show a highly dysregulated expression in ovarian cancers of all histological subtypes, with many studies showing either up- or down-regulation of HOX genes. Their functions have not been completely elucidated but they undoubtedly have a critical function in oncogenesis. They have been shown to have an important role in cell survival and proliferation, cell motility, differentiation of tissue-specific lineage and coordinating the immune response to tumour cells. The pathways by which they act are likely to be numerous and require further elucidation. The differential expression of HOX genes may well prove to be a useful diagnostic or prognostic biomarker, especially given their easy availability in tumour tissue, biological fluids such as ascites and circulating tumour cells in blood. Evidence is also accumulating that they could be used as a predictive biomarker of drug response, particularly platinum sensitivity.

The significance of HOX genes is underscored by the development of drugs targeted against them, particularly HXR9, which has shown anticancer efficacy both in vitro and in vivo, and also synergy with commonly-used first line chemotherapy agents, such as the platinums.

## Figures and Tables

**Table 1:** HOX gene expression. Table 1 shows the 39 HOX genes and indicates which are commonly over or under expressed in ovarian cancer cell lines or tissues relative to normal ovarian surface epithelium. Grey boxes are HOX genes that do not exist. (↑ = overexpressed, ↓ = underexpressed).

HOX Gene	A	B	C	D
1				↑
2		↑		
3				
4	↑	↑		
5		↑		
6		↑	↓	
7	↑	↑		
8		↑		
9	↑			
10	↑			
11	↑			
12				
13	↑	↑	↑	↑
